# Supplementary material for: Content-rich biological network constructed by mining PubMed abstracts
Source: BMC Bioinformatics. 2004 Oct 8;5:147. doi: 10.1186/1471-2105-5-147 (PMC528731; doi:10.1186/1471-2105-5-147)
Supplement: Additional File 2 — The original results of the above study (non-essential files are deleted to keep the file size under the limit set by BMC bioinformatics). [file 1471-2105-5-147-S2.bz2 › chilibotAdditionalFile2/dip20/469ID9660793E10525/html/CCL13_CXCR3.html]

 


 **CCL13** and **CXCR3** 
  
Found 13 abstracts in PubMed, retrieved 13.  
 

 What does Google say? 
 PDF only 
| .edu only 

---

**Interactive relationship** (e.g. stimulation, inhibition, etc)

**Inhibitory relationship**- Identification of potent, selective non peptide CC chemokine receptor 3  [ **CXCR3** ]  antagonist that inhibits eotaxin, eotaxin 2, and monocyte chemotactic protein 4  [ **CCL13** ]  induced eosinophil migration.  Ref: 10969084 J Biol Chem, 2000
- I TAC, Mig, and IP10  [ **CXCR3** ]  compete for the binding of eotaxin to CCR3 bearing cells and inhibit migration and Ca 2 changes induced in such cells by stimulation with eotaxin, eotaxin 2, MCP 2 monocyte chemottractant protein 2, MCP 3, MCP 4  [ **CCL13** ] , and RANTES regulated on activation normal T cell expressed and secreted.  Ref: 11110785 J Biol Chem, 2001
**Neutral relationship**- Surprisingly, several CC chemokines, particularly eotaxin and MCP 4  [ **CCL13** ] , also compete with moderate affinity for the binding of IP10  [ **CXCR3** ]  to  **CXCR3** .  Ref: 9660793 J Biol Chem, 1998
- CC chemokine receptor 3  [ **CXCR3** ]  is also activated by other promiscuous ligands such as RANTES and monocyte chemotactic protein 4  [ **CCL13** ] .  Ref: 11286622 J Invest Dermatol, 2001
- Several CC chemokines including eotaxin CCL 11, eotaxin 2 CCL 24, RANTES CCL 5, and monocyte chemotactic protein 3 MCP 3, CCL 7 and 4 MCP 4  [ **CCL13** ] , CCL 13  [ **CCL13** ]  are potent eosinophil chemotactic and activating peptides acting through CC chemokine receptor 3  [ **CXCR3** ]  CCR3.  Ref: 10969084 J Biol Chem, 2000

**Non-interactive relationship** (e.g. studied together, co-existance, homology, etc.)

- Eosinophil chemotactic chemokines eotaxin, eotaxin 2, RANTES, monocyte chemoattractant protein 3 MCP 3, and MCP 4  [ **CCL13** ] , and C C chemokine receptor 3  [ **CXCR3** ]  expression in bronchial biopsies from atopic and nonatopic Intrinsic asthmatics.  Ref: 10570327 J Immunol, 1999
- From among the 13 chemokines examined, MCP 3, MCP 4  [ **CCL13** ] , RANTES, and IP 10  [ **CXCR3** ]  were detected in the disc from the idiopathic scoliosis, and MCP 3, MCP 4  [ **CCL13** ] , RANTES, IP 10  [ **CXCR3** ] , MIG, and MGSA alpha were detected in the infected or herniated discs.  Ref: 12131709 Spine, 2002
- The RNA was reverse transcribed, and the resultant cDNA was amplified by PCR using primer pairs specific to the CXC chemokines IL 8, MGSA alpha, IP 10  [ **CXCR3** ] , MIG, the CC chemokines MCP 1, MCP 2, MCP 3, MCP 4  [ **CCL13** ] , MIP 1alpha, MIP 3alpha, RANTES, STCP 1, the C chemokine lymphotactin, and the glyceraldehyde phosphate housekeeping gene.  Ref: 12131709 Spine, 2002
